# Characterization of Litigation After Tympanoplasty and Mastoidectomy in the United States

**DOI:** 10.7759/cureus.81192

**Published:** 2025-03-25

**Authors:** Alyssa D Reese, Lauren A DiNardo, Soumya Gupta, Kristina F Powers, Samuel Colca, Michele M Carr

**Affiliations:** 1 Otolaryngology, University at Buffalo Jacobs School of Medicine and Biomedical Sciences, Buffalo, USA; 2 Otolaryngology, University of Rochester Medical Center, Rochester, USA; 3 Otolaryngology - Head and Neck Surgery, Montefiore Medical Center, New York, USA; 4 Otolaryngology - Head and Neck Surgery, Eastern Virginia Medical School (EVMS) Medical Group at Old Dominion University, Norfolk, USA

**Keywords:** litigation outcomes, mastoidectomy, medical malpractice, tympanomastoidectomy, tympanoplasty

## Abstract

Introduction

Tympanoplasty and mastoidectomy are common procedures performed by otolaryngologists that can result in complications for which patients may seek compensation. Medical malpractice case analyses may offer insight into how clinicians can avoid risk and improve patient satisfaction. We aimed to comprehensively characterize litigation after mastoidectomies and tympanoplasties in the United States.

Methods

The Westlaw Campus Research legal database was searched for all available court decisions associated with claims of medical malpractice after tympanoplasty and/or mastoidectomy in the United States between 1975 and 2022. Information on the plaintiffs’ relationships to the patients, patient characteristics, states where the procedures took place, specialties of the defendants, allegations, surgical and postoperative complications, and adjudicated case outcomes was collected. Descriptive statistics were calculated.

Results

Fifteen cases that took place between 1976 and 2019 involving tympanoplasty (n = 2 (13.3%)), mastoidectomy (n = 4 (27.7%)), both tympanoplasty and mastoidectomy (n = 8 (53.3%)), and revision mastoidectomy (n = 1 (6.7%)) were reviewed. Most of the cases involved patients who were >18 years old (13/15 (86.7%)) and female (8/15 (53.3%)). An otolaryngologist was listed as a defendant in almost all cases (14/15 (93.3%)), and a hospital, surgery center, or otolaryngology practice was listed in 7/15 (46.7%) cases. The most common (13/15 (86.7%)) reason given for medical malpractice was negligent technique. Resulting injuries included facial nerve injury (5/15 (33.3%)), brain injury or infection (3/15 (20.0%)), death (2/15 (13.3%)), and a retained foreign body (1/15 (6.7%)). Most cases (11/15 (73.3%)) were ruled in favor of the defendant, and most (12/15 (80.0%)) were affirmed on appeal.

Conclusions

There are few claims of medical malpractice after tympanoplasty or mastoidectomy in the United States that are decided in court. Injury to the facial nerve or brain was associated with the plaintiff winning in the cases analyzed in this study.

## Introduction

Tympanoplasty and mastoidectomy are common otologic procedures that were introduced over 100 years ago [1]. These and various ancillary procedures are used to treat a wide array of otologic conditions, including chronic otitis media and cholesteatoma [2]. Earlier reports estimated that 30,000-60,000 mastoidectomies were performed per year in the United States [2], and analyses of ambulatory surgery databases of several states showed that tympanoplasty was the most common adult otologic procedure [3].

Tympanoplasty and mastoidectomy require advanced anatomical knowledge and precise surgical technique because the tympanic membrane and mastoid are proximal to important neurological and vascular structures. Common complications of these procedures include hearing loss, facial nerve injury, changes in taste, tinnitus, and persistent infection. Such outcomes can be the basis for litigation for medical malpractice. The aim of this study was to provide an updated analysis of the reasons for medical malpractice claims after mastoidectomy, tympanoplasty, and tympanomastoidectomy in the United States and to determine the outcomes of litigation. In doing so, we hope to identify potential areas where improvement may reduce physician risk of involvement in medical malpractice cases.

## Materials and methods

Study design

The online legal database Westlaw Campus Research (Thompson Reuters, New York, NY, USA) was retrospectively reviewed for jury verdict reports and summary statements from all state and federal legal cases related to tympanoplasty or mastoidectomy in the United States. This study was deemed to be exempt from review by the University at Buffalo Institutional Review Board because it was a retrospective review of a publicly available database.

Case selection

The Westlaw Campus Research database was searched using the terms (malpractice OR negligence) AND (tympanoplasty, mastoidectomy, mastoid, OR tympanomastoidectomy). Opinions written between January 1975 and May 2022 in which medical negligence or malpractice claims occurred specifically in relation to the tympanoplasty, mastoidectomy, or tympanomastoidectomy were included. Duplicated cases and cases with limited information were excluded from our study. Additionally, Eighth Amendment violations that involved the cruel and unusual punishment of prisoners were not included. The Westlaw Campus Research database does not contain information on cases settled or dropped before going to court. Therefore, cases that were settled out of court were excluded from our study.

Data collection

The following data were collected from each case: gender and age of the patient, relationship of the plaintiff to the patient, state where the tympanoplasty or mastoidectomy took place, procedures performed, specialty of the defendant(s), reason(s) for the alleged medical malpractice or medical negligence, and case outcome. If an appeal occurred, available data were collected. The verdict of the appeal was either affirmed (i.e., the appellate court agreed with the lower court decision), reversed (i.e., the appellate court disagreed with the lower court decision), affirmed and remanded, reversed and remanded (i.e., the appellate court overturned the lower court decision and returned it to the lower court for further litigation), or vacated (i.e., the appellate court did not provide a ruling and returned the case to the lower court).

Analysis

IBM SPSS Statistics for Windows, Version 27.0 (Released 2020; IBM Corp., Armonk, NY, USA) was used to calculate descriptive statistics. The numbers and percentages of patients or cases are expressed accordingly.

## Results

**Figure 1:**
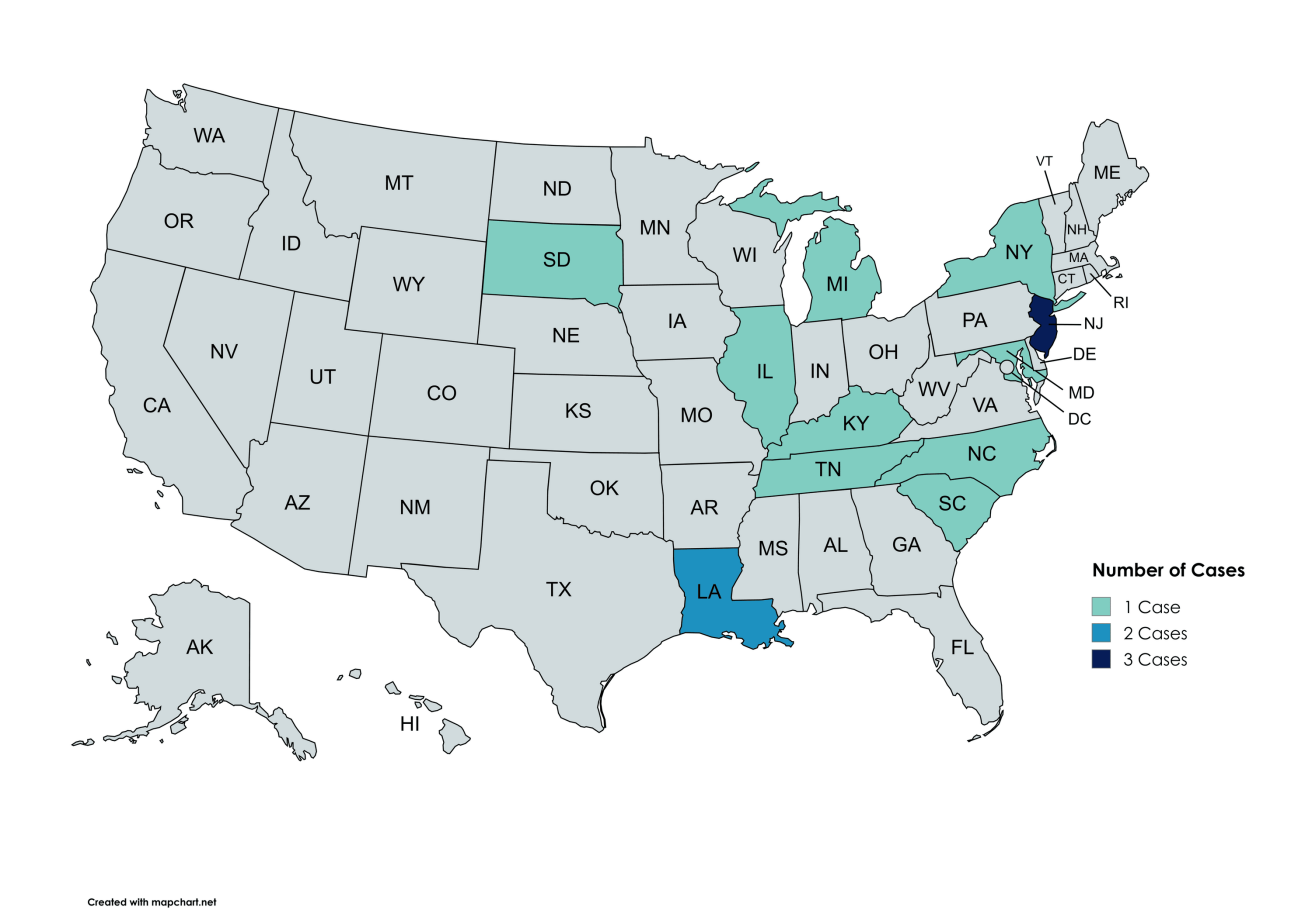
Geographic distribution of cases by state

Fifteen cases between 1976 and 2019 were identified and included in the analysis. Most patients involved in the cases were older than 18 years (Table 1); the two pediatric patients were 18 months and one year old. The cases were most often initiated by the patient and were related to a tympanomastoidectomy. The procedures were performed in several US states (Figure 1), but one was performed in England by a US Air Force physician.

**Table 1:** Patient characteristics

Characteristic	No. (%) of patients (n = 15)
Sex	
Female	8 (53.3)
Male	7 (46.7)
Age (year)	
>18	13 (86.7)
0-18	2 (13.3)
Status	
Alive	13 (86.7)
Deceased	2 (13.3)
Plaintiff’s relationship with the patient	
Patient	8 (53.3)
Patient and parent(s)	1 (6.7)
Significant other	2 (13.3)
Patient and significant other	4 (26.7)
Procedure	
Tympanoplasty	2 (13.3)
Mastoidectomy	4 (26.7)
Tympanomastoidectomy	8 (53.3)
Revisionary mastoidectomy	1 (6.7)

Almost every case listed an otolaryngologist as the defendant (Table 2) and claimed negligent technique as the reason for medical malpractice (Table 3). Informed consent medical malpractice most commonly involves the lack of a discussion of complications such as potential nerve damage.

**Table 2:** Specialty involvement classifications of the defendants

Specialty of defendant	No. (%) of cases (n = 15)
Otolaryngology	14 (93.3)
Hospital, surgery center, or otolaryngology practice	7 (46.7)
Anesthesiology	1 (6.7)
Pediatrics	1 (6.7)

**Table 3:** Reasons cited by plaintiffs for claim of medical malpractice

Reason	No. (%) of cases (n = 15)
Negligent technique	13 (86.7)
Consent process	7 (46.7)
Failure to diagnose	3 (20.0)
Failure of surgery	5 (33.3)
Wrongful death	1 (6.7)
Postoperative management	1 (6.7)

A summary of the cases is provided in Table 4. There was one case where the surgeon knowingly performed surgery on both ears, even though the consent form incorrectly stated surgery was to be performed on the left ear and right ear surgery was discussed during the informed consent process. The resulting injuries claimed in the 15 cases included postoperative facial nerve dysfunction (n = 5 (33.3%)), brain injury or infection (n = 3 (20%)), and a retained foreign body (n = 1 (6.7%)). There were two deaths: one was the result of brain injury and subsequent meningitis following a mastoidectomy, and the other was the result of the administration of nitrous oxide in place of oxygen during a tympanoplasty. Most cases (11/15 (73.3%)) were ruled in favor of the defendant, and most (12/15 (80.0%)) were affirmed on appeal.

**Table 4:** Summary of cases FD, failure to diagnose; FS, failure of surgery; IC, informed consent; M, mastoidectomy; NT, negligent technique; PM, postoperative management; RM, revision mastoidectomy; T, tympanoplasty; TM, tympanomastoidectomy; WD, wrongful death

Year	Procedure	Allegation	Resulting injury	Initial verdict	Result of appeal
1979	TM	NT, IC	Facial nerve injury	Case ongoing	
1986	TM	NT, IC	Facial nerve injury	For defendant	Affirmed
1987	TM	NT, IC, FS, PM	Facial scarring, failure to remove all of the cholesteatoma	For defendant	Affirmed for hospital defendant; remanded for physician defendant
1993	TM	NT, IC	Facial nerve injury	For defendant	Affirmed
1993	TM	NT, FD, FS	Recurrent cholesteatoma	Case dismissed	Affirmed
2008	TM	NT, IC, FD	Facial NT, hearing loss, loss of balance, and tinnitus (FD)	For plaintiff	Affirmed ($1.8 million in damages)
2018	TM	FD	Hearing loss	For defendant	Affirmed
2019	TM	NT, FS	Hearing loss	For defendant	Affirmed
1976	M	NT	Facial nerve injury	For defendant	Affirmed
1992	M	NT	Sigmoid sinus brain bleed, loss of vision	For defendant	Affirmed
2004	M	NT, FS	Meningitis, death	For defendant	Remanded
2007	M	NT	Retained foreign body, chronic infections	For defendant	Affirmed
2004	RM	NT, IC	Brain injury	For plaintiff	Remanded
1983	T	NT, IC, FS	Incorrect consent form, surgery on both ears without proper consent, failure to treat cholesteatoma	For defendant	Affirmed
1984	T	WD	Anesthesia complication, death	For defendant	Affirmed for three surgeons and hospital; remanded for the anesthesiologist

## Discussion

According to a 2017 survey, 78% of otolaryngologists have been sued at least once during their career [4]. Although only 10% of malpractice allegations within the field of otolaryngology are otology related [5], an evaluation of the extent to which specific otologic procedures contribute to such malpractice claims can help educate physicians and reduce the burden of these claims [6]. Two studies focused on malpractice litigation in otologic procedures were published in 2013 [7,8]. The present study examines claims related specifically to tympanoplasty and mastoidectomy to date.

We found that negligence was the most common reason for malpractice, consistent with reports of litigation involving otologic, otolaryngologic, and surgical procedures [5-7]. A negligent technique is established when a physician deviates from the standard of care and the deviation causes harm [9]. For a plaintiff to recover damages in a legal case, there is an onus to prove that a defendant had a duty to care for the patient, there was a breach of duty (or deviation from the standard of care), harm occurred, and negligence was the cause of the harm [10]. Tympanomastoidectomies are difficult because of the anatomical complexity of the region and the proximity of cranial nerves, inner and middle ear structures, and the brain. Thus, the provision of the standard of care does not ensure that injury to the patient will not occur. This may explain why most of the cases in this study were ruled in favor of the defendant and affirmed upon appeal.

Claims of negligent technique in many cases cite surgical complications and injuries as contributing factors. The complications that were most common in the cases reviewed in the present study were facial nerve injury during surgery and hearing loss due to failure to treat or diagnose cholesteatomas. This aligns with complications from mastoidectomies in cases reported by Blake et al. [8], in which facial nerve injury and hearing loss were claimed in four and two of the six cases, respectively, and with those from tympanoplasties in cases reported by Ruhl et al. [7]. Ruhl et al. [7] reported that 26/58 (45%) and 22/58 (38%) of all otologic surgery malpractice cases alleged hearing loss and facial nerve injury, respectively. Intraoperative facial nerve monitoring (IOFNM) can be used to help reduce facial nerve injury, specifically with the use of an attachable magnetic nerve stimulator [11,12]. Gidley et al. [11] surveyed members of the American Neurotology Society, American Otological Society, and American Society of Pediatric Otolaryngology, and the majority agreed that IOFNM was indicated for most otology or neurotology procedures. Further, 86.1% of these physicians reported receiving training in IOFNM [12]. However, reimbursement for the procedure remains difficult to obtain [11,12].

Brain injury and infection were alleged in 3/15 (20%) cases in our study. In two of these cases, the original verdict was remanded by a higher court on appeal. This suggests that the critical nature of such injuries complicates judicial decision-making. The severity of these complications may place an otolaryngologist at risk for malpractice litigation. The possibility of these complications should be addressed with the patient, as informed consent may help to avoid potential claims that cite both informed consent and negligent technique when such complications occur.

A lack of informed consent, including the lack of discussion of potential complications, was cited in multiple cases in our study (33.3%). This is similar to the report by Blake et al. [8], in which 31.9% of the malpractice cases involving otologic procedures claimed a lack of informed consent. Thorough discussion is necessary prior to obtaining consent for difficult otologic surgeries such as tympanoplasty and mastoidectomy. Patients should understand the anatomical complexity of the area and be well informed about the potential surgical complications. These discussions should be documented in the medical record to protect the physician if there is litigation. The physician should only note the complications discussed because automatically populating the electronic medical record could lead to discrepancies that could be used against the physician in court [13].

The outcomes of pediatric cases of malpractice are often in favor of the patient and plaintiff [14], possibly because of the severity of the complications. The two pediatric cases in our study were initiated because of the failure to diagnose a condition requiring tympanomastoidectomy. However, the outcomes of both cases were in favor of the defendants who were pediatricians and a US Air Force physician. The reasons for this are not clear, but the cases illustrate the need for otolaryngologists and pediatricians to seriously address concerns of hearing loss and to provide appropriate referrals for pediatric patients.

Limitations

A limitation of this study was the use of a single database. The Westlaw Campus Research database compiles federal and state cases that have been published and made publicly available. Although federal and state appellate court opinions and federal lower court opinions are included, lower or trial court decisions for most states may not be [15]. Any cases not brought to the state or federal court are thus excluded. Therefore, our analysis may not reflect the outcomes of all tympanoplasty and mastoidectomy cases with malpractice claims. Of the cases that were included in our study, there was no standardization of information provided, and the extent of reporting varied between cases. Legal documentation pertaining to medical procedures and medical history is not as specific or structured as typical medical records: the specialty of expert witnesses was typically not recorded, and the type of medical practice of the defendant otolaryngologist was not always documented. In addition, the analysis was performed on a small sample size so we were unable to make statistical comparisons.

## Conclusions

Few cases of litigation after tympanoplasty or mastoidectomy in the United States go to trial. When medical malpractice cases arise, the rulings frequently favor the involved medical professional. However, litigation can be successful for cases involving injury to the facial nerve or brain. To avoid litigation, potential complications of these procedures should be discussed thoroughly before surgery, and the use of IOFNM should be considered.
